# Interventions on health equity in the future

**DOI:** 10.1093/eurpub/ckac129.436

**Published:** 2022-10-25

**Authors:** TA Eikemo

**Affiliations:** Center for Global Health Inequality Research, Norwegian University of Science and Technology, Oslo, Norway

## Abstract

A final reflection on the importance of evaluation aspects will be proposed at the end of the workshop.

